# Use of aromatase inhibitor in a girl with peripheral precocious puberty

**DOI:** 10.1186/1687-9856-2013-S1-P74

**Published:** 2013-10-03

**Authors:** CY Lee, SW Cheng, LK Lee

**Affiliations:** 1 Department of Paediatrics & Adolescent Medicine, Caritas Medical Centre, Hong Kong

## 

This girl presented with problem of vaginal bleeding at 6 year 2 month old. There was no medication history. Physical examination showed that she was tall with height 122.7 cm (97^th^ percentile) and weight was 23.1 Kg (75-90^th^ percentile). Early pubertal sign was present with Breast stage 2 and pubic hair stage 1. She did not have skin pigmentation and has no bone deformity or fracture. Investigations suggested peripheral precocious puberty with grossly elevated serum estradiol 2293 pmol/L with suppressed LH < 0.2 IU/L and FSH < 0.2 IU/L, which also failed to rise in the Gonadotrophin stimulation test. Bone age was advanced to 7 to 8 year old. Pelvic ultrasound showed a 3.6 cm cystic lesion in left ovary and the uterus size was prominent with endometrial echoes. Skeletal survey did not show bony lesion. The clinical diagnosis was peripheral precocious puberty from autonomous functioning ovary cyst.

The girl showed progression with recurrent vaginal bleeding and further breast development. To stop further vaginal bleeding and prevent later compromised height, Letrozole, an aromatase inhibitor, was commenced at dose of 1.25 mg daily. Breast size regressed and serum estradiol dropped to nadir of <73 pmol/L. The ovary cyst resolved 2 months after starting Letrozole but was again detected after stopped treatment at 7 years old. The girl went into true puberty around when she approached 9 year old with progression of breast development and growth spurt. The height at last follow up at 10- year 10 month old was 154 cm (97^th^ percentile) and has not yet started menarche. Repeated ultrasound at 8 year 9 month old showed that the ovary cyst again resolved.

The case demonstrated that the potent, long-acting aromatase inhibitor Letrozole is effective in arresting sexual precocious puberty resulting from autonomous functioning ovary cyst. The girl goes into normal puberty and attains growth spurt. Further follow up is required to monitor normal menstruation pattern.

